# Supplementary material for: A set of multi-entry identification keys to African frugivorous flies (Diptera, Tephritidae)
Source: Zookeys. 2014 Jul 24;(428):97–108. doi: 10.3897/zookeys.428.7366 (PMC4143993; doi:10.3897/zookeys.428.7366)
Supplement: Supplementary material 4 — Key to Capparimyia [file zookeys-428-097-s004.zip › SF4_ZooKeys_key to Capparimyia/lucid_player/help/help/trees_and_lists_in_lucid3.htm]

Trees and Lists


# Trees and Lists

In some Lucid3 keys, features
and entities may be arranged into hierarchical trees, with sub-features
nested within features and sub-entities nested within entities. For example,
a key to palm species may have features arranged using a morphological
hierarchy (with leaf, flower and fruit features nested under appropriate
headings), and entities arranged into a taxonomic hierarchy (with species
nested within genera etc.).

If the features and entities in a key are arranged into trees, you may
choose to display the features and entities as trees or lists by selecting
the appropriate tab at the bottom of the *Features* and *Entities*
panels.

Features and Entities displayed as a tree

Features and Entities displayed as a list

**Note** that some operations
and modes in Lucid3 work only with lists. For example, Sort
Best and Ranked Match Mode require that the key is in List View, as
only lists can be sorted or ranked.
